# Aldose Reductase (AR) Mediates and Perivascular Adipose Tissue (PVAT) Modulates Endothelial Dysfunction of Short-Term High-Fat Diet Feeding in Mice

**DOI:** 10.3390/metabo13121172

**Published:** 2023-11-24

**Authors:** Daniel J. Conklin, Petra Haberzettl, Kenneth G. MacKinlay, Daniel Murphy, Lexiao Jin, Fangping Yuan, Sanjay Srivastava, Aruni Bhatnagar

**Affiliations:** 1 Center for Cardiometabolic Science, University of Louisville, Louisville, KY 40202, USA; petra.haberzettl@louisville.edu (P.H.); daniel.murphy@hsc.utah.edu (D.M.); lexiao.jin@louisville.edu (L.J.); sanjay.srivastava@louisville.edu (S.S.); aruni@louisville.edu (A.B.); 2 Division of Environmental Medicine, Department of Medicine, University of Louisville, Louisville, KY 40202, USA; fangping.yuan@louisville.edu; 3 School of Medicine, University of Louisville, Louisville, KY 40202, USA; kgmack01@gmail.com; 4 Christina Lee Brown Envirome Institute, Louisville, KY 40202, USA

**Keywords:** adiposity, cardiovascular disease, endothelium, leptin, lipid-derived aldehydes, nitric oxide, obesity, polyol pathway

## Abstract

Increased adiposity of both visceral and perivascular adipose tissue (PVAT) depots is associated with an increased risk of diabetes and cardiovascular disease (CVD). Under healthy conditions, PVAT modulates vascular tone via the release of PVAT-derived relaxing factors, including adiponectin and leptin. However, when PVAT expands with high-fat diet (HFD) feeding, it appears to contribute to the development of endothelial dysfunction (ED). Yet, the mechanisms by which PVAT alters vascular health are unclear. Aldose reductase (AR) catalyzes glucose reduction in the first step of the polyol pathway and has been long implicated in diabetic complications including neuropathy, retinopathy, nephropathy, and vascular diseases. To better understand the roles of both PVAT and AR in HFD-induced ED, we studied structural and functional changes in aortic PVAT induced by short-term HFD (60% kcal fat) feeding in wild type (WT) and aldose reductase-null (AR-null) mice. Although 4 weeks of HFD feeding significantly increased body fat and PVAT mass in both WT and AR-null mice, HFD feeding induced ED in the aortas of WT mice but not of AR-null mice. Moreover, HFD feeding augmented endothelial-dependent relaxation in aortas with intact PVAT only in WT and not in AR-null mice. These data indicate that AR mediates ED associated with short-term HFD feeding and that ED appears to provoke ‘compensatory changes’ in PVAT induced by HFD. As these data support that the ED of HFD feeding is AR-dependent, vascular-localized AR remains a potential target of temporally selective intervention.

## 1. Introduction

Endothelial dysfunction (ED)—*sine qua none* of atherosclerosis, erectile dysfunction, hypertension, and peripheral vascular diseases—is an early and predictive marker of chronic cardiovascular disease (CVD) [1]. ED is also prominent in diabetes, and, in fact, because of its many vascular complications, diabetes is more recently recognized as a primary risk factor for CVD [2]. Notably, obesity, a risk factor for diabetes, and the quantity of specific adipose depots such as visceral and perivascular adipose tissue (PVAT) associate more strongly with markers of CVD than do either subcutaneous adiposity or body mass index (BMI) [3]. Recent studies suggest that a localized relationship exists within the vascular wall between the vascular components (smooth muscle cells and endothelium) and the surrounding PVAT that contributes to vascular complications such as ED and contributes to an increase in other systemic diabetes markers (e.g., insulin resistance and hyperglycemia) [4,5]. As changes in the PVAT surrounding blood vessels contribute to ED and the subsequent development of and progression to CVD, research into the precise spatio-temporal changes in adipose and vascular function is needed to unravel how these changes contribute to ED. As such, biochemical, transcriptional, structural, and functional changes in the PVAT are documented in studies of animal models under a variety of pre-diabetic conditions, including HFD feeding and leptin deficiency [6,7]. Collectively, these studies provide evidence to indicate that changes in PVAT contribute to alterations in vascular function, including the onset and perpetuation of ED [8,9].

For over half a century, the role of aldose reductase (AR) in the cardiovascular and other system complications of diabetes has been investigated [10]. Numerous AR inhibitors (ARIs) have been tested in clinical trials, but there remains limited clinical availability in the U.S., excepting ARIs internationally for the treatment of neuropathic foot pain—a debilitating disease of diabetes [11]. The search for effective ARIs against a variety of diabetic complications as well as other outcomes persists [12]. Thousands of pre-clinical studies have been published that demonstrate ARI efficacy in animal models of diabetes, with a vast majority of these studies being in Type I diabetic models (i.e., streptozotocin-induced), wherein insulin deficiency induces severe hyperglycemia and body wasting [13]. ARI treatment dramatically reduces cardiovascular-related complications in these models, indicating a primary role of AR in mediating excessive glucose reduction in the development of vascular complications, including ED and vasculitis [14,15]. However, as 95% of human cases of diabetes are Type II diabetes, and these cases are associated highly with obesity [16], it is important to understand the role of AR in animal models of Type II diabetes (T2D, e.g., leptin-deficient obese rodents, high-fat diet (HFD) feeding) that has been less completely investigated. Moreover, the evaluation of AR as a potential target of pharmacological intervention in T2D could provide new information on potential treatment strategies [15].

It is well known that compounds other than glucose are the preferred substrates of AR. In fact, endogenous AR substrates include lipid peroxidation-derived aldehydes (acrolein, HNE, and ONE), their glutathione conjugates such as GS-propanal and GS-4-hydroxynonanal, as well as carnosine-aldehyde adducts [17,18]. AR-mediated catalysis of these aldehydes occurs at lower, more physiological concentrations than for the reduction of glucose (K_m_s: μM vs. mM), and thus, AR’s physiological role may well be to limit the direct toxicity of highly reactive electrophiles [19]. Regardless of AR’s varying substrate affinity and despite the many studies of ARI in hyperglycemia, to date, no study has examined the role of AR in changes induced by short-term HFD feeding in PVAT adiposity and vascular function. Moreover, it is unclear how these changes relate to alterations in systemic obesity and vascular complications. Thus, this study was undertaken to assess the early changes in structure and function of PVAT and vascular function under short-term HFD feeding in male wild type (WT) and AR-null mice. Whole-body AR-deficient mice were used as a test of AR dependence in HFD feeding to better understand the pathogenic sequelae driving early vascular dysfunction in diet-induced obesity [20].

## 2. Materials and Methods

Materials: If not otherwise stated, analytical-grade reagent chemicals were purchased from MilliporeSigma (St. Louis, MO, USA).

Mice: C57BL/6J mice (WT, wild type; The Jackson Laboratories, Bar Harbor, ME, USA) and aldose reductase-null (AR-null; in-house colony) mice [21] were housed in pathogen-free, AAALAC-accredited facilities under 12 h:12 h light/dark cycle with controlled temperature and humidity. Mice were treated according to APS’s Guiding Principles in the Care and Use of Animals and all protocols were approved by University of Louisville IACUC.

For this study, (AR-null) mice bred on a C57Bl/6 background and wild type (WT) (C57Bl/6) male mice were maintained in a temperature-controlled room (22 °C) on a normal chow (NC) diet composed of 59% kcal from carbohydrates, 29% kcal from protein, and 12% kcal from fat (LabDiet 5010, PMI Nutrition, St. Louis, MO, USA) after weaning. At 8 or 18 weeks of age, mice were either switched to a high-fat (HF) diet composed of 60% kcal from fat (lard-based), 20% kcal from protein, and 20% kcal from carbohydrates (Research Diets INC, New Brunswick, NJ, USA) for 4 weeks or maintained on an NC diet for 4 weeks. During the feeding period, the body weight of the animals was monitored weekly. After the mice fasted, their final body weights were measured, and the mice were injected with sodium pentobarbital (150 mg/kg, 0.1 mL).

Fat Composition: Mouse body fat percentage was analyzed using a dual-energy X-ray absorptiometry (DEXA) scan following pentobarbital injection using a Lunar PIXImus (GE Healthcare, Waukesha, WI, USA) bone density scanner. Each mouse head was excluded from the final composition measurements [22].

**Scheme 1:**
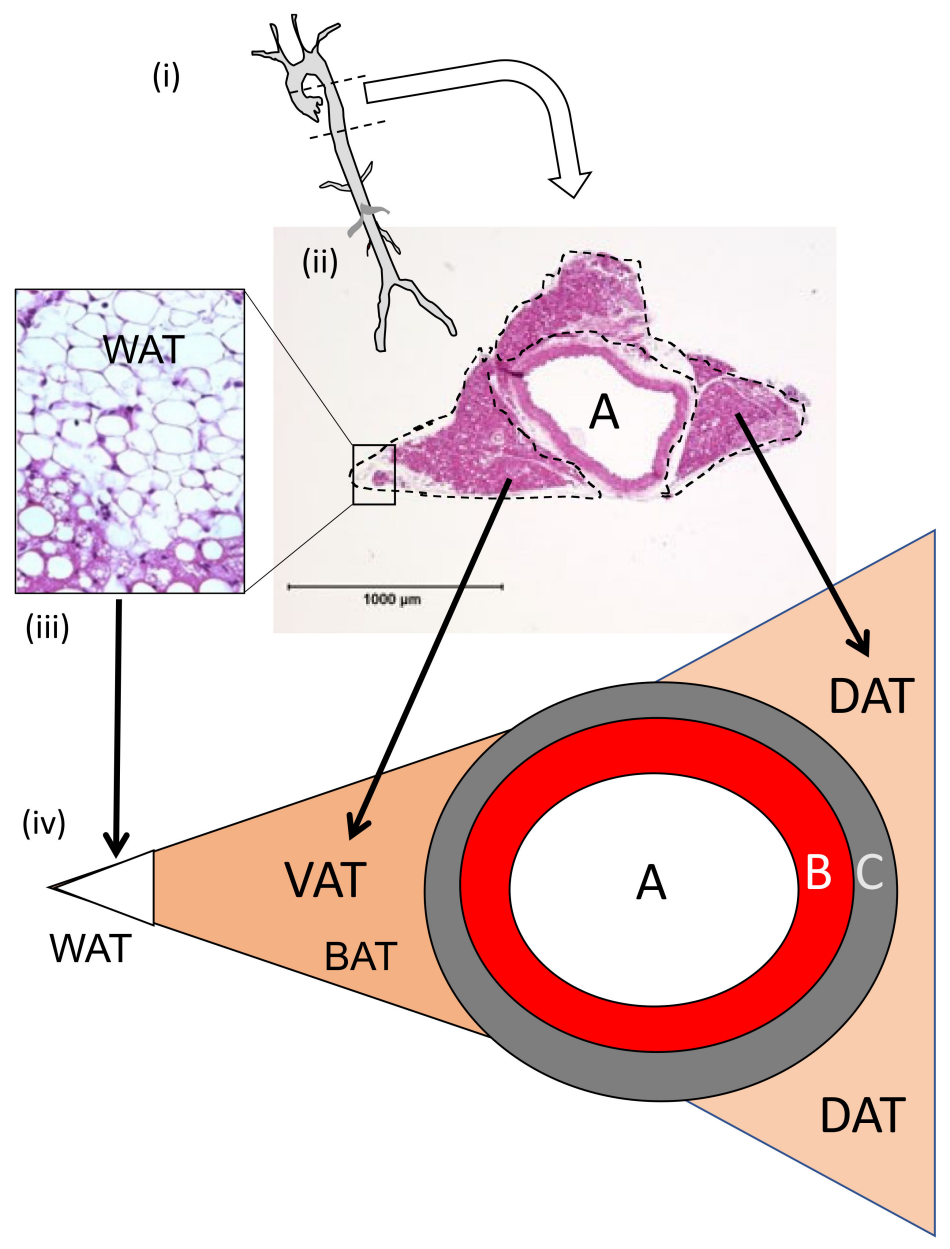
(**i**) Morphometry and function of perivascular adipose tissue (PVAT) were analyzed in sections of the thoracic aorta. (**ii**) The representative thoracic aorta cross-section shows that the aorta is surrounded by a triangular set of 2 adipose masses: a ventral adipose tissue (VAT) and a dorsal adipose tissue (DAT), which is composed of a right and left DAT connected by a short isthmus. Both VAT and DAT are largely composed of brown adipose tissue (BAT), with smaller clusters of white adipose tissue (WAT) containing enlarged adipocytes (**iii**). (**iv**) Cartoon of an aortic cross-section. Formalin-fixed, aortic cross-sections were stained with hematoxylin-eosin (H&E), and images were acquired with light microscopy (magnification: 100×). Size-stamped images were imported into ImageJ software for quantification of the following measures: aortic lumen area (A; μm^2^); aortic medial area (B; μm^2^); aortic adventitial area (C; μm^2^); dorsal PVAT area (DAT; μm^2^); ventral PVAT area (VAT; μm^2^); white adipose tissue (WAT) cluster number and area; and adipocyte number and area (μm^2^). Dashed lines represent areas of VAT and DAT, respectively. Brown-colored areas represent BAT. Scale line = 1 mm.

Histology: A 5 mm length of aorta was obtained from the distal thoracic aorta (Scheme 1). Aortic sections were placed in processing cassettes and fixed in 10% neutral buffered formalin (Leica Microsystems; Wetzlar, Germany). Histology was performed on formalin-fixed, paraffin-embedded aortic cross-sections stained with hematoxylin and eosin (H&E). Images were obtained at ≈100× using Spot advanced image capture software (SPOT Imaging Solutions, Sterling Heights, MI, USA). Image analysis was performed using NIH image processing and analysis in java (ImageJ version 1.x) software (NIH free software 1.x). Luminal, adventitial, and medial areas were measured on aortic cross-sections using ImageJ drawing tools (Scheme 1).

Dorsal and Ventral Adipose Tissue: Perivascular adipose tissue (PVAT) is divided into ventral adipose tissue (VAT) and dorsal adipose tissue (DAT). All area measurements were obtained using ImageJ. These measurements were performed by outlining the outer edge of VAT and DAT using ImageJ drawing tools, i.e., tracing the adventitia–PVAT border and completing measurements around the outside of VAT and DAT.

White Adipocyte Measurements: White adipocytes and their clusters were distinguished from BAT by the larger cell size with little staining of the cells. Clusters were defined as having ≥5 adjacent white adipocytes (Scheme 1). Areas of clusters were measured from the cross-sections using ImageJ drawing tools as described above, and DAT and VAT were measured using ImageJ. Clusters were typically found on the periphery of PVAT, usually surrounding blood vessels. The area of individual adipocytes in these clusters was also measured using ImageJ drawing tools, and adipocytes in the clusters were enumerated manually.

Histopathology and Immunofluorescence: Formalin-fixed, paraffin-embedded aorta and PVAT tissue blocks from WT and NC (*n* = 3), AR-null and NC (*n* = 3), WT and HFD (*n* = 3), and AR-null and HFD (*n* = 4) mice were sectioned at 4 µm thickness, placed on glass slides, and processed through deparaffinization and rehydration. H&E staining was performed with hematoxylin (Fisher Scientific, Waltham, MA, USA, 220-100and Eosin-Y (Fisher Scientific, 220–104), and H&E images were captured using an Olympus IX71 microscope equipped with CellSens Standard software 1.x.

For immunofluorescence (IF) staining, deparaffinized and rehydrated sections from experimental groups were blocked with 0.1% Triton X-100 and 1% BSA in PBS (pH 7.4) at RT for 30 min to reduce nonspecific binding. Slides were incubated overnight with primary antibodies in PBS with 1% BSA at 4 °C. The following primary antibodies were used for leptin (PA1-051, Invitrogen, Carlsbad, CA, USA, 1:100 dilution) and aldose reductase (SC-166918, Santa Cruz Biotech, Dallas, TX, USA, 1:50 dilution). On the next day, after slides were washed with PBS, sections were incubated with Alexa Fluor 488 Donkey anti-Rabbit secondary IgG (Invitrogen, 1:400) and Alexa Fluor Plus 594 Donkey anti-Mouse secondary IgG (Invitrogen, 1:200) in PBS with 1% BSA and 10% normal donkey serum at RT for 90 min. Slides were counterstained with DAPI for nuclear labeling. Sections stained with fluorophore-labeled secondary antibodies only (no primary antibodies) served as negative controls. IF Images were acquired by Nikon Eclipse TI microscope with NIS-Elements software 1.x. All slides were imaged under equal conditions of intensity and duration.

Individual sections of 3 different mice per WT and NC and WT and HFD groups were examined for the intensity of AR immunofluorescence. AR-null groups were not examined. Five equally sized, representative areas of the aortic media were selected for each mouse. Each area was selected to avoid overlap with adventitia, endothelium, PVAT, and anomalous structures such as vascular branches, coagulated blood, or obvious section defects (e.g., folds, clumping of stain, etc.). Individual RGB images were imported to ImageJ, and a threshold level was selected based on iterative comparison. Once selected, a threshold level was applied uniformly to all sections, and the % area and mean fluorescence intensity (MFI) values were provided by ImageJ. A similar assessment process was also performed for DAPI staining (nuclear, blue) to check whether differences in cell density/number occurred.

Vascular reactivity: Thoracic aortas were isolated, and vascular reactivity was assayed as described previously [23]. Briefly, one ~3–4 mm ring per mouse was hung on stainless steel hooks in 15-mL water-jacketed organ baths in physiological salt solution (PSS) bubbled with 95% O_2_ and 5% CO_2_ at 37 °C. The composition of PSS was (in mM): NaCl, 130; KCl, 4.7; MgSO_4_·7H_2_O, 1.17; KH_2_PO_4_, 1.18; NaHCO_3_, 14.9; CaCl_2_, 2.0; glucose, 5.0; pH 7.4. Rings (~1 g loading tension) were contracted with 100 mM potassium solution (2 times) and then with cumulative concentrations of phenylephrine (PE; 0.1 nM–10 µM). PE-precontracted rings were relaxed with cumulative concentrations of acetylcholine (ACh; 0.1 nM–10 µM) or of sodium nitroprusside (SNP; 0.1 nM–10 µM) to measure endothelium-dependent or -independent relaxation, respectively. Vessel contraction was quantified as mg tension, active stress (in mN/mm^2^), or normalized as a percentage of the maximum PE contraction [24]. Relaxation was calculated as a percentage reduction in PE-induced tension. The effective concentration producing a 50% response (EC_50_) was assessed by normalizing cumulative concentration responses to 100%, plotting the response vs. the log [molar]_agonist_, and interpolating the EC_50_ [23].

Statistical analysis: Data are reported as mean ± SEM. For comparing two groups, an unpaired Student’s *t*-test was used. Comparisons between multiple groups were performed by One-Way ANOVA on ANOVA on Ranks followed by a Bonferroni or Holm–Sidak post hoc test where appropriate (SigmaStat ver. 12, SPSS, Chicago, IL, USA). Statistical significance was accepted at *p* < 0.05.

## 3. Results

### 3.1. Short-Term High-Fat Diet Feeding Increases Body and PVAT Adiposity

To understand how short-term HFD feeding alters aortic structure and function, we fed male WT and AR-null mice NC or HFD for 4 weeks, beginning at either 8 or 18 weeks old.

#### 3.1.1. Body Weight and Adiposity

**Figure 1:**
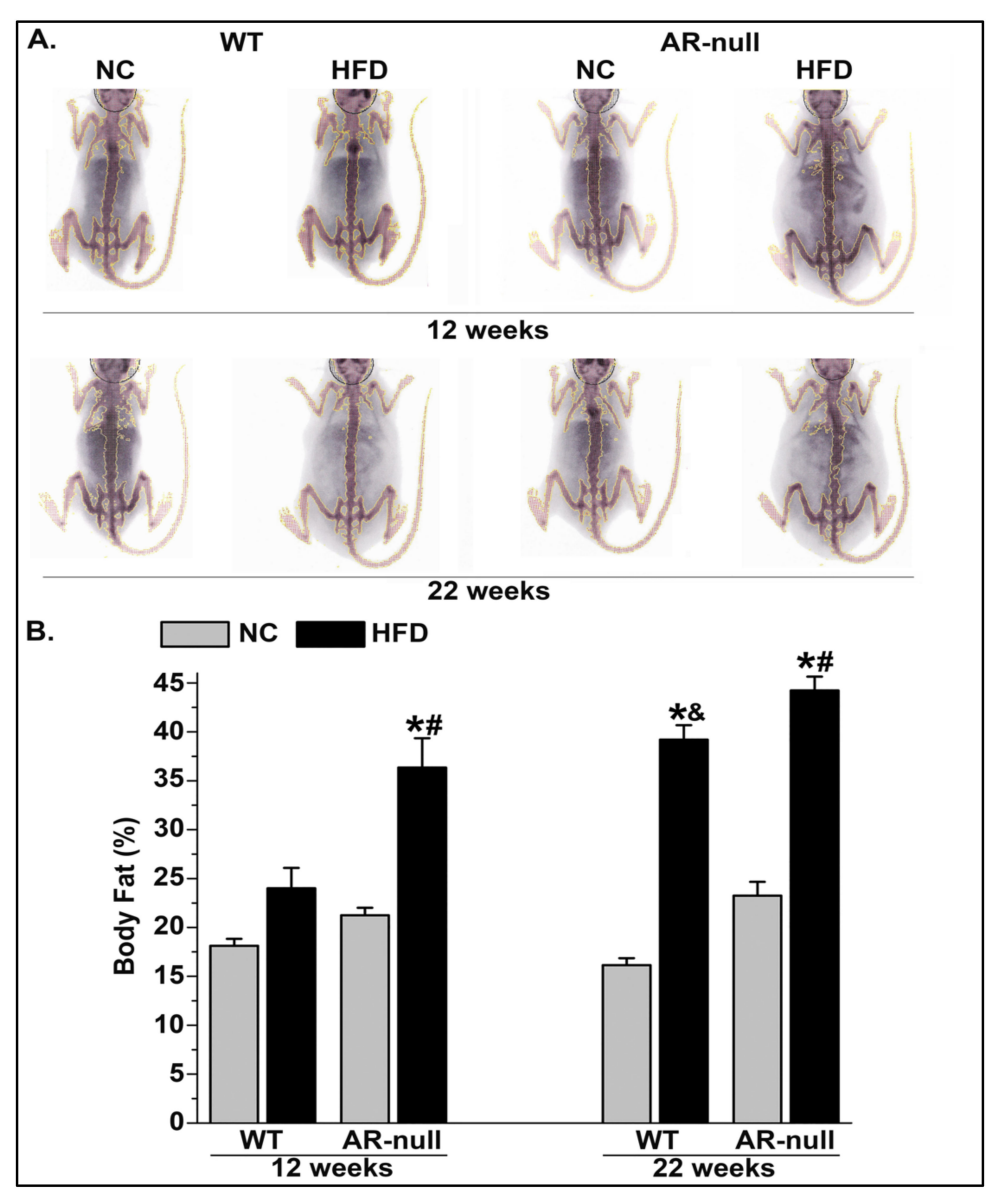
Effects of high-fat diet (HFD) on whole-body adiposity measured by dual-energy X-ray absorptiometry (DEXA) scan in male WT and AR-null mice. (**A**) Representative DEXA images of WT and AR-null mice fed normal chow (NC) or HFD for 4 weeks, either between the ages of 8 and 12 weeks or 18 and 22 weeks prior to DEXA. (**B**) Summary data of DEXA scan-derived % body fat following 4 weeks of feeding of NC or HFD. Values = means ± SE (*n* = 4–8). *, *p* < 0.05 vs. age-matched NC control; #, *p* < 0.05 vs. age- and diet-matched WT control; and & *p* < 0.05 vs. genotype- and diet-matched 12-week control.

Feeding mice HFD for 4 weeks increased the fasting blood glucose levels, body weight, and gonadal fat mass (Table 1) as well as the percentage of body fat of both male WT and AR-null mice as measured by DEXA scan with an age effect (Figure 1). Although HFD feeding significantly increased the body fat percentage in both WT and AR-null mice when mice were started on the HFD at 18 weeks of age, only AR-null mice had significantly increased body fat percentage when HFD feeding started at 8 weeks of age (Figure 1A). In WT and AR-null mice fed HFD for 4 weeks from 18 to 22 weeks old, the body fat percentage was approximately double that of the age-matched mice fed NC (Figure 1B). Additionally, the gonadal fat pad mass (as % of BW) increased by HFD feeding both in WT (2-4X) and AR-null (3X) mice, independent of age (Table 1). Similarly, fasting blood glucose increased by HFD feeding both in WT and in AR-null mice (10–20%), with greater levels observed in older mice (Table 1).

#### 3.1.2. Perivascular Adipose Tissue (PVAT) Morphometry: Effects of HFD

**Figure 2:**
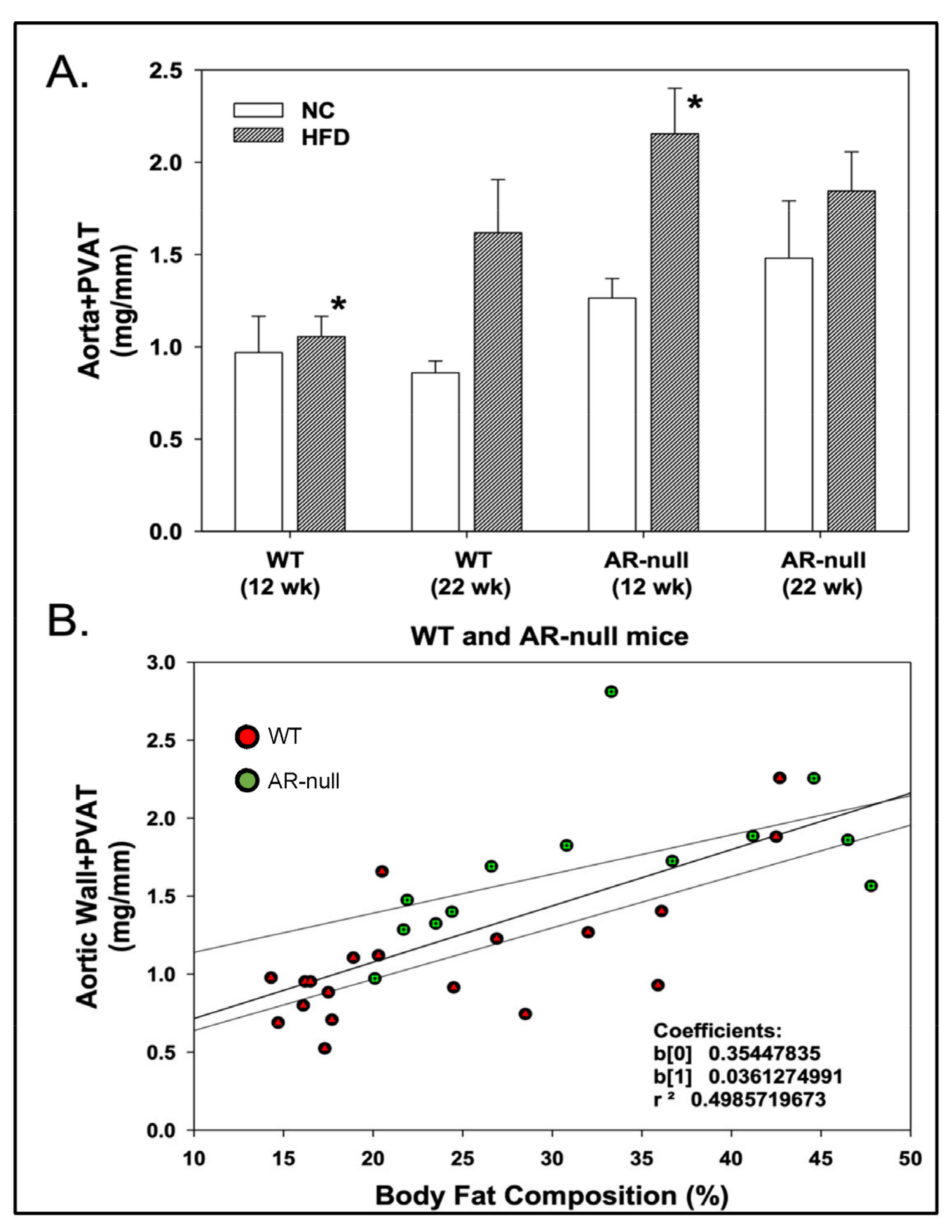
Effects of high-fat diet (HFD) on thoracic aorta perivascular adipose tissue (PVAT) mass (mg/mm) and whole-body adiposity in male WT and AR-null mice. (**A**) Summary data of thoracic aorta plus PVAT mass following feeding of normal chow (NC) or HFD for 4 weeks between the ages of 8 to 12 weeks and 18 to 22 weeks of age in male WT and AR-null mice prior to collection of thoracic aorta for gravimetric analysis. (**B**) Regression analysis of % body fat by DEXA scan vs. thoracic aorta plus PVAT mass (mg/mm). Each circle represents 1 mouse (red = WT; green = AR-null). Values = means ± SE (*n* = 4–8). *, *p* < 0.05, WT and HFD vs. age-matched AR-null and HFD.

PVAT and Aorta Analysis: To quantify the relationship between body fat percentage and aortic PVAT, PVAT and aorta mass (mg) were normalized to aortic length (mm) (Figure 2A). HFD feeding slightly enhanced the normalized PVAT and aorta mass, yet it was only significantly greater in AR-null mice fed HFD (from 8 to 12 weeks of age) compared with age- and diet-matched WT mice (Figure 2A). A significant positive correlation (r^2^ ≅ 0.5) between body fat percentage and normalized PVAT and aorta mass was observed similarly in both WT and AR-null mice (Figure 2B).

**Figure 3:**
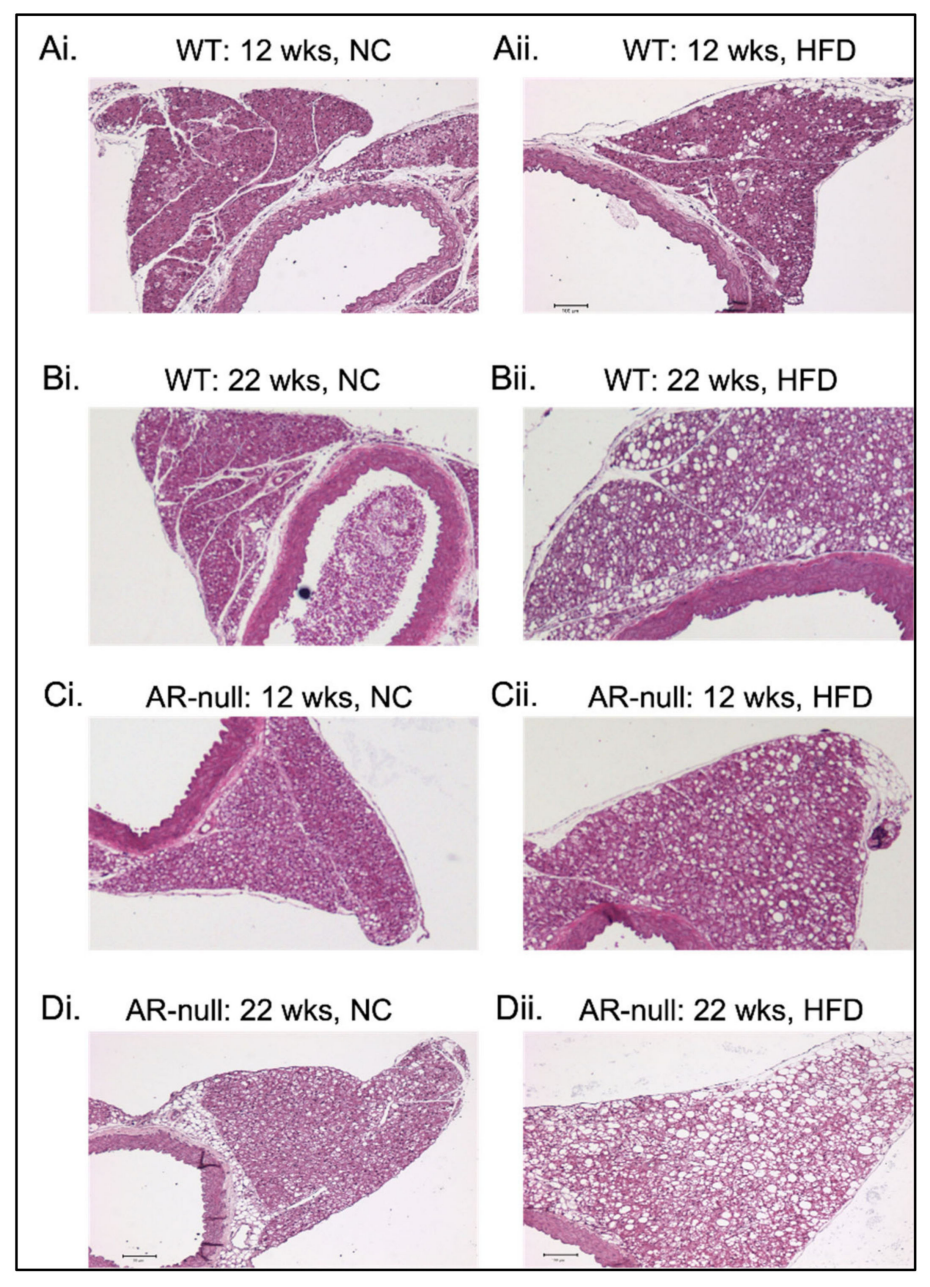
Effects of high-fat diet (HFD) on mid-thoracic aorta perivascular adipose tissue (PVAT) in male WT and AR-null mice. Representative images of H&E-stained aortic PVAT cross-sections of WT (**A**,**B**) and AR-null (**C**,**D**) mice fed normal chow (NC) or HFD for 4 weeks either between the ages of 8 and 12 weeks (**A**,**C**) or 18 and 22 weeks (**B**,**D**) of age prior to the collection of the aorta for structural and functional analyses. Where present, scale bar = 100 µm.

To understand how increased body fat percentage influenced PVAT size and composition, histological and morphometric analyses were performed on fixed aortas with intact PVAT attached. In H&E-stained cross-sections, HFD feeding for 4 weeks appeared to increase the area of the PVAT, accompanied by the occurrence of larger (white) adipocytes and clusters thereof (Figure 3), indicating that the PVAT changes from a brown fat to a white fat phenotype. Although HFD feeding appeared to increase the appearance of a white adipocyte tissue (WAT) phenotype in all groups, it was most noticeable in the AR-null mice fed HFD from 18- to 22-weeks of age (Figure 3(Dii)).

PVAT Area Measurements: To quantify the apparent changes in PVAT area and composition induced by HFD feeding, dorsal adipose tissue (DAT), ventral adipose tissue (VAT), and total PVAT areas were measured using ImageJ according to the Scheme 1. In general, 4 weeks of HFD feeding increased DAT, VAT, and total PVAT areas, with typical increases in the overall PVAT area of +40–50% that were independent of age and genotype (Table 2). Notably, the PVAT area appeared larger in NC-fed AR-null mice when compared with age-matched WT mice (Table 2).

Adipose Tissue Areas with White Adipose Tissue (WAT) Characteristics, Number, and Area Analyses: Because HFD reliably enhanced the PVAT area, we asked whether the increase was a function of changes in WAT. Although areas with clusters of white adipocytes were a fraction of the total PVAT area, these areas were increased by short-term HFD feeding, independent of age and genotype. However, HFD feeding induced the strongest increase in these clusters (4×) in WT mice (Table 2). Consistent with the increased areas of WAT in WT mice, both the cell number and cell area of adipocytes with a WAT character were increased (Table 2). Again, lesser changes were observed in AR-null mice fed HFD, in part, because baseline numbers and areas of white adipocytes were greater in AR-null mice fed an NC diet when compared with diet- and age-matched WT mice (Table 2).

Aorta Cross-sectional Measurements: The aortic cross-sectional area was measured using ImageJ. In general, aortic cross-sectional areas grew only modestly, if at all, with HFD feeding in both WT and AR-null mice (Table 2). No significant differences in aortic cross-sectional areas were observed across age, diet, or genotype (Table 2).

### 3.2. Aortic Function with and without PVAT: Effects of Age, Genotype, and Diet

A critical determinant of normal blood vessel function is the presence of a healthy endothelium. To assess the endothelium function, we isolated a segment of the mid-thoracic aorta with and without intact PVAT and evaluated contractility and relaxant function via isometric myography in vitro [25].

#### 3.2.1. Relaxation Responses

Agonist-induced relaxation was stimulated by either an endothelium-dependent or -independent pathway.

**Figure 4:**
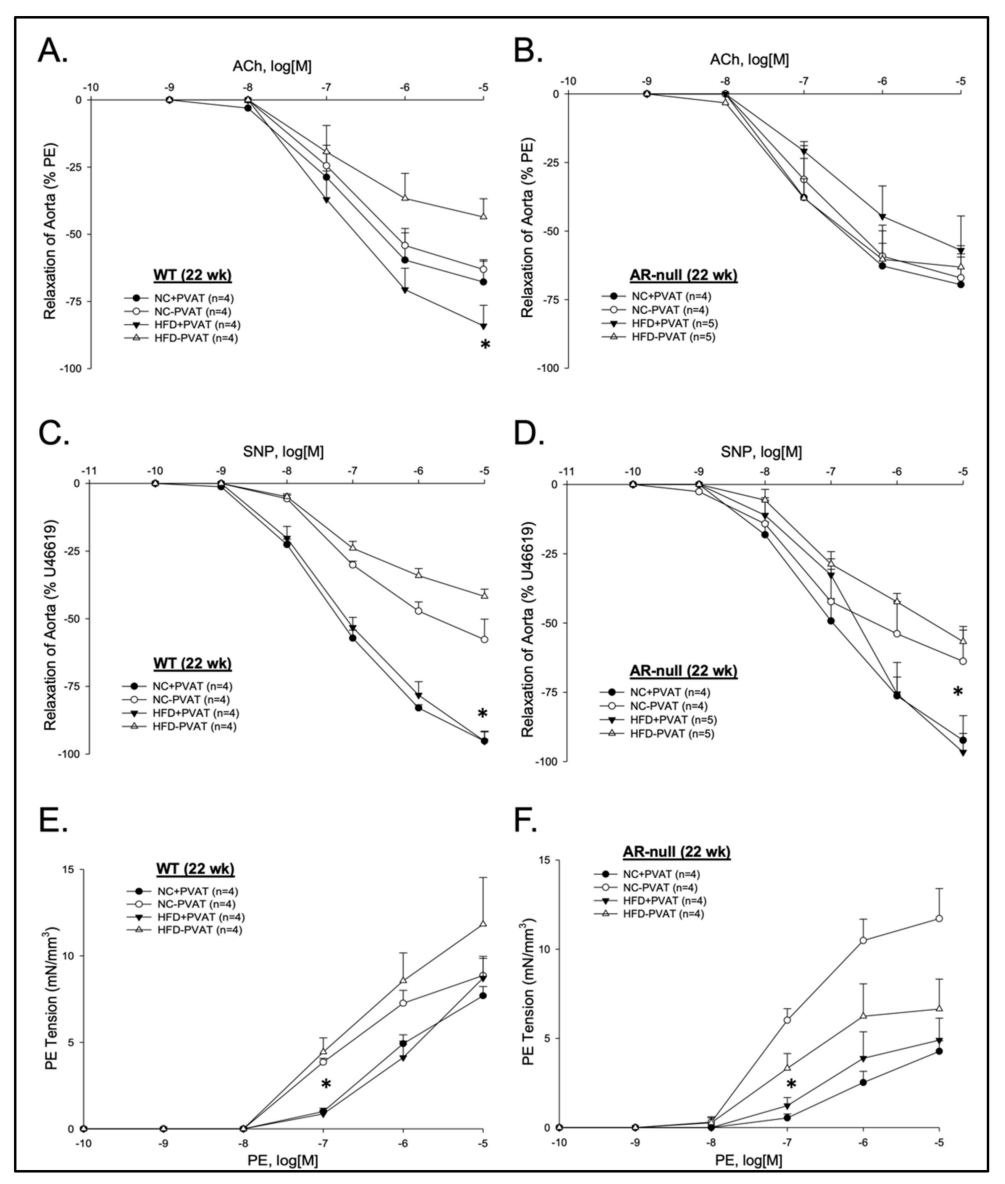
Effects of short-term high-fat diet (HFD) on thoracic aorta function without and with perivascular adipose tissue (+PVAT). Male C57BL/6 WT and AR-null mice were fed either normal chow (NC) or HFD for 4 weeks from the age of 18 to 22 weeks old. Following euthanization, the thoracic aorta was isolated, and the functional properties of the aorta with and without intact PVAT were measured by isometric myography. Acetylcholine (ACh) was used to assess aortic endothelium-dependent relaxation efficacy (% relaxation) in phenylephrine (PE)-precontracted aortic rings of WT (**A**) and AR-null (**B**) mice. Sodium nitroprusside (SNP; nitric oxide donor vasorelaxant) was used to assess endothelium-independent relaxation in U46619-precontracted aorta of WT (**C**) and AR-null (**D**) mice. The efficacy (mN/mm^3^) of concentration-dependent, phenylephrine (PE)-induced contractions was measured in aortic rings of WT (**E**) and AR-null (**F**) mice. Values = mean ± SE (*n* = 4–5 mice per group); *, *p* < 0.05 vs. matched control PVAT.

Endothelium-dependent (ACh) Responses: To assess the diet, age, and genotype effects on the endothelium-dependent pathway of relaxation, we added increasing cumulative concentrations of ACh in PE-precontracted aortic segments either with or without PVAT. ACh induced similar concentration-dependent relaxations independent of age or the presence of PVAT, except for differences observed in the aorta of WT mice fed HFD. Notably, the aorta of WT mice fed HFD with intact PVAT relaxed significantly better than its matched aortic segment without PVAT (Figure 4A; Supplementary Figure S1A). This PVAT-dependent effect was not observed in the aorta of age- and diet-matched AR-null mice (Figure 4B and Figure S1B), indicating that the effect was both diet- and AR-dependent. In addition, in PVAT-free aortic preparations of 22-week-old WT mice, HFD-feeding significantly diminished ACh-induced relaxation (endothelial dysfunction)—an effect not observed in the aortas of any other group (Figure 4A).

Endothelium-independent (SNP) Responses: To assess the endothelium-independent pathway of relaxation, we added increasing cumulative concentrations of the NO donor, SNP, in PE-precontracted aortic segments either with or without PVAT. SNP induced concentration-dependent relaxations that were independent of age, diet, or genotype yet notably enhanced in aortas with PVAT compared with aortas without PVAT in 22-week-old mice (Figure 4C,D). SNP-induced relaxations were similar in the aortas with and without PVAT of 12-week-old mice (Supplementary Figure S1C,D).

#### 3.2.2. Contractile Responses

Phenylephrine (PE) induced concentration-dependent contractions that were decidedly more sensitive and typically stronger in the aortas without PVAT compared with aortas with PVAT (but not at the highest PE concentration) (Figure 4E,F and Figure S1E,F). This pattern was independent of age, diet, or genotype, reflecting the general and dominate anti-contractile function of PVAT. However, 4 weeks of HFD appeared to lessen the difference in the contractions of the aorta with and without PVAT by decreasing the contractility of the aorta in general (Figure 4E,F and Figure S1E,F).

#### 3.2.3. Immunofluorescence Localization of AR and Leptin in Aorta and PVAT

**Figure 5:**
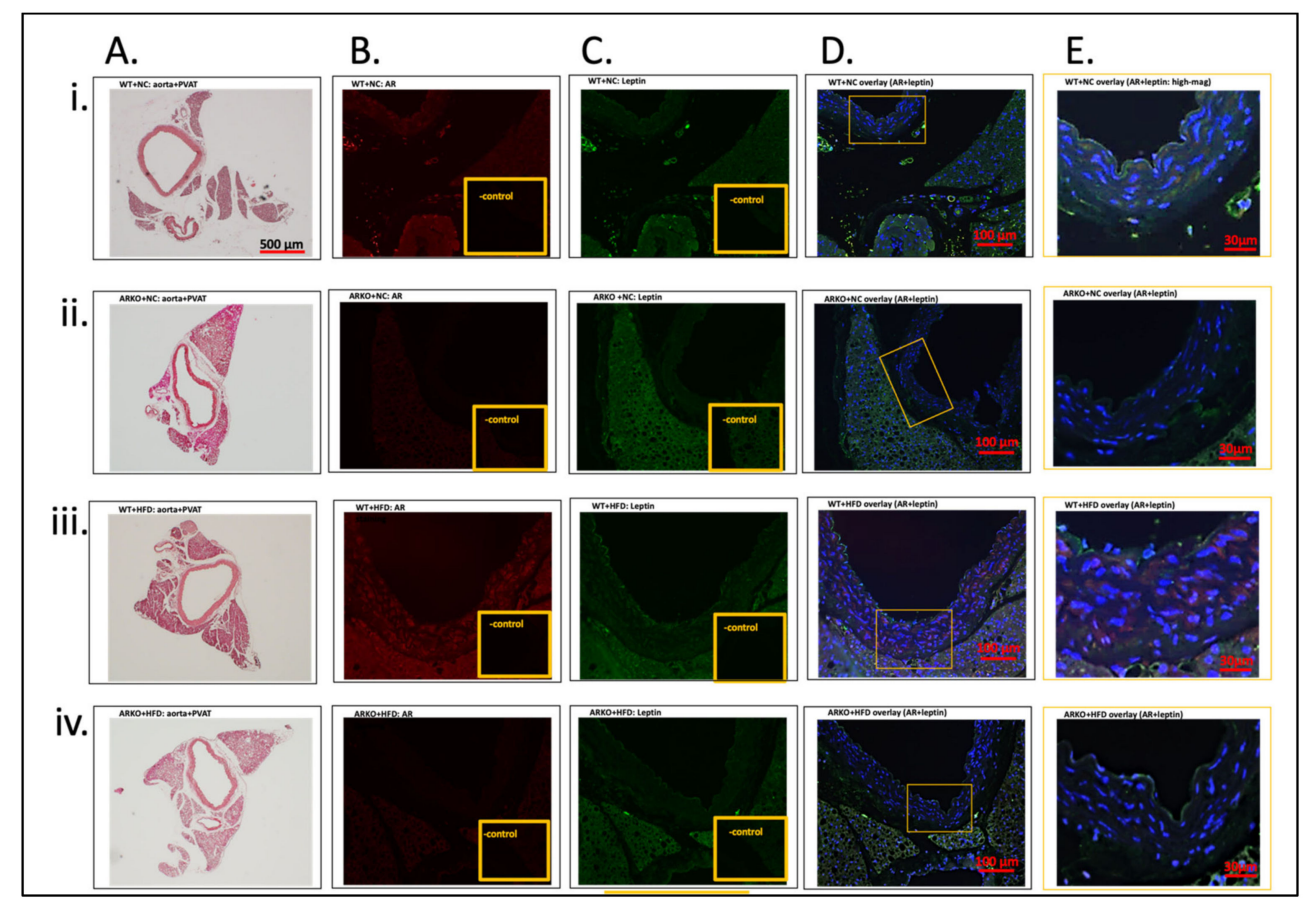
Representative cross-sectional images of thoracic aorta with intact perivascular adipose tissue (PVAT) stained with the following: (**A**) H&E (40×; scale bar = 500 µm); (**B**) anti-aldose reductase (AR; red; 200×; inset box, negative control lacks primary AR antibody); (**C**) anti-leptin (green; 200×; inset box, negative control lacks primary leptin antibody); and, (**D**) DAPI (blue, nuclear) and overlay of (**B**,**C**) (200×; scale bar = 100 µm); and (**E**) higher magnification of inset box shown in (**D**) (600×; scale bar = 30 µm). (**i**) WT and NC, 22 wks; (**ii**) AR-null and NC, 22 wks; (**iii**) WT and HFD, 22 wks; and (**iv**) AR-null and HFD, 22 wks.

To better understand the relationship between AR, PVAT, and endothelial dysfunction, we performed immunofluorescent localization of AR and leptin in the aortic wall with intact PVAT. Leptin was chosen as a marker of PVAT adipocytes because leptin increases in blood in proportion to increased white adipose, and leptin mRNA is increased in murine aortic PVAT with exogenous stress in mice. AR is well known to be expressed in both vascular smooth muscle cells and endothelial cells. Additionally, AR is increased in VSMC in response to hyperglycemia in vivo or high glucose in vitro. As expected, positive AR staining was present in the aortic media (VSMC) of the WT and NC group (Figure 5B(i)), with noticeable enhanced red fluorescence both in the aortic wall and in PVAT of the WT and HFD group (Figure 5B(iii)). To support the specificity of the AR antibody, appropriately, no red staining was observed either in AR-null mice (Figure 5B(ii,iv)) or in the negative controls of the WT sections that lacked an addition of the primary AR antibody (see inset, Figure 5B(i,iii)). Conversely, positive leptin staining (green) was present diffusely in the PVAT of all four groups with occasional bright “hot” spots (Figure 5C(i–iv)), yet minimal staining was observed in the aortic wall, except for some noticeable endothelium staining in the WT and HFD group (Figure 5C(iii)). As the purpose of the staining was to assess the potential co-localization of these two proteins, we examined the staining overlay of AR with leptin (Figure 5D(i–iv)) and further magnified a region of interest, where the PVAT and aortic wall were in close opposition (Figure 5E(i–iv)). This proved most revealing in the WT and HFD group, wherein strong red aortic wall staining (AR positive) was shifted to a more yellowish/orange hue with the co-presence of green staining (leptin positive), including in the endothelium (Figure 5E(iii)). Nuclear staining (blue, DAPI), especially in the VSMC of media of the WT and HFD group, appeared enlarged and more disorganized (Figure 5E(iii)) compared with the other three groups (Figure 5E(i,ii,iv)).

Because AR immunofluorescence in the VSMC (media of the aorta) appeared enhanced by HFD feeding, we quantified both the area and MFI of AR staining in WT and NC and WT and HFD. Unsurprisingly, we had a significant increase in WT and HFD vs. WT and NC both in the AR-stained area (as % of area assessed) (WT and HFD: 12.97 ± 4.00; WT and NC: 3.91 ± 0.48; *n* = 3, 3, *p* = 0.05) and in AR MFI (WT and HFD: 201,095 ± 22,863; WT and NC: 65,382 ± 11,245 AU; *n* = 3, 3, *p* = 0.002). There, however, was no significant change in the DAPI-stained area (nuclear) in WT and HFD vs. WT and NC (WT and HFD: 21.45 ± 1.52; WT and NC: 18.92 ± 2.60; *n* = 3, 3).

## 4. Discussion

Because obesity continues to be a global healthcare issue that increases the risk of developing both Type II diabetes and CVDs, there is a need to better understand how obesity influences the cardiovascular system and pathogenesis [5,26]. Notably, increased visceral and PVAT adiposity, but not subcutaneous adiposity, is most associated with an increased CVD risk. However, the mechanisms by which visceral adiposity affects CVD risk are incompletely known, yet more recent studies indicate the involvement of local pro-inflammatory fat depots such as PVAT in pathogenic cardiovascular changes [27]. The results of our current study in an animal model of short-term HFD feeding support the general idea that PVAT grows rapidly with HFD feeding and that PVAT changes in its structure and function in as little as a few weeks. In the context of short-term HFD feeding, the physiological changes in PVAT appear compensatory in nature—perhaps to offset the development of ED—and our data support this relatively new idea [28]. Additionally, the short-term HFD feeding-induced ED is AR-dependent—a novel observation, although not totally unexpected given previous studies. Moreover, the systemic absence of AR appears to promote slightly greater basal fat accumulation under normal chow (NC) as well as under HFD feeding conditions. Yet, these HFD-induced changes in systemic and PVAT adiposity and in increased (20%) blood glucose did not promote ED in AR-null mice, which further supports the primary role of ED in initiation of vascular dysfunction and corresponding compensatory PVAT change. Notably, in HFD-fed AR-null mice, the generally anticontractile function of PVAT was unchanged (see Figure 4).

Aldose reductase (AR) is a widely expressed aldehyde and glucose metabolizing enzyme that functions in the reduction of cytoxic aldehydes formed during lipid peroxidation. Glucose is an incidental substrate of AR, and the metabolism of glucose via the AR-catalyzed polyol pathway generates sorbitol, and sorbitol is associated with secondary diabetic outcomes, especially the well-known vascular complications [29]. For half a century, the inhibition of AR and the polyol pathway, in general, was pursued as a therapeutic target for managing the complications of diabetes [15]. Additionally, an unexpected side effect of ARI is that it blocks the late phase of ischemic preconditioning in a preclinical model [30]. In principle, the genetic deficiency of AR in rodent models (AR-null) of diabetes supports the central role of AR in complications of hyperglycemia, but it also reveals that AR plays critical roles in the renal concentration of urine, with the specific mechanistic details still to be elucidated [21]. Our current study expands the potential physiological roles of AR to include adipocyte differentiation and deposition; although this was a serendipitous finding, it is not entirely novel [31].

More critically, we show for the first time that short-term HFD-feeding-induced ED is AR-dependent. We suspect this outcome results from AR localized in the endothelium or the VSMC (or both), although we did not discriminate between these possibilities. Immunofluorescent staining supports that AR was induced in VSMC (and perhaps other cells) by short-term HFD feeding, which is plausible as AR is induced by hyperglycemia along with other stimuli, and HFD feeding increases fasting blood glucose by approximately 20% in both WT and AR-null mice (see Table 1). How AR contributes to ED with HFD feeding, however, is unclear, but perhaps it is via its catalytic function, as ARIs are shown to inhibit deleterious changes in endothelial cells and VSMC due to hyperglycemic and inflammatory stimuli alike [32,33,34,35,36]. However, other organs/tissues are also affected by HFD feeding, such as peripheral nerves [37], subcutaneous adipose tissue [31], and the liver [38], in an AR-dependent manner. Each of these target organs may also contribute in meaningful ways to our model. This supposition remains to be assessed in more in-depth mechanistic studies using the short-term HFD feeding model.

Another notable finding in our present study is that the PVAT appears to rapidly and physiologically compensate for an injured/deficient endothelium [28]. This change to a more anticontractile state is not present in AR-null mice, regardless of age or diet, and thus, these data support the inference that compensation is in response to ED and not a product of AR deficiency, diet, and/or the growth of PVAT with HFD feeding. In fact, PVAT expanded similarly (but not exactly the same) in both WT and AR-null mice fed HFD for 4 weeks. Despite quantifying regional differences in PVAT as we defined it (i.e., ventral adipose tissue, VAT; dorsal adipose tissue, DAT), we cannot discern at the gross structural level whether compensatory changes are uniformly delivered from VAT, DAT, or both. The results of a previous study of PVAT in the aorta of naïve NC-fed mice suggest that both VAT and DAT act in an anti-contractile manner, in part through the release of leptin as well as other factors [25]. Although it may be valuable to identify the specific component(s) of this compensatory response, it is noteworthy that several models of chronic HFD feeding in different mammals (mice, rats, and pigs) uniformly find that PVAT becomes pro-inflammatory and deleterious to the underlying vasculature, especially the endothelium, suggesting that the “compensatory pathway” is at best temporary (i.e., “too much of a good thing”). In the Type II diabetes model of Goto-Kakizaki rats, the aortic PVAT is pro-inflammatory, pro-oxidant, and pro-contractile, and it overexpresses AR compared with Wistar rat control [39]. The role of AR in that model was untested. Nonetheless, in our model, short-term HFD-feeding in WT (but not AR-null) mice elicited robust PVAT physiological compensation to offset ED in the short term.

Chronic HFD feeding leads to increased obesity and elevated circulating leptin levels, and ultimately to ‘leptin resistance’, both centrally and locally [4,6,40], which may be related to cardiovascular consequences [41]. Because white adipose tissue (WAT) grew in the cluster area, the number of WAT cells, and the WAT cell area in the predominately brown adipose tissue (BAT) of thoracic PVAT with 4 weeks of HFD feeding, we stained the aortic wall for leptin. Although positive leptin staining was uniformly present in PVAT (with few brightly stained cells), there was little evidence that it was dramatically increased in this time frame by HFD. Perhaps this is not surprising given that the increased area of WAT amounted to <2.5% of the total PVAT, yet the WAT was organized into clusters that may well be important in local vascular wall changes due to the local release of adipokines such as leptin [40,42,43]. Although this may be plausible, AR-null mice had just as much (and more) WAT in PVAT and systemically than WT mice did after 4 weeks of HFD, yet AR-null mice did not have ED. Importantly, AR-null mice did not have a PVAT ‘compensatory’ function either. Thus, the evidence for a dominant role of the whitening of PVAT or too much leptin in this short-term, HFD-induced, AR-dependent ED is lacking in this study.

Despite the fact that these observations reveal critical roles of AR and PVAT in the ED of HFD feeding, this study has several limitations. We only used male mice, and it is possible that female mice under similar conditions may have altered quantitative outcomes due to protective hormones in female mice, and thus, sex dependence should be pursued as well. We also used a genetically deficient model of AR rather than using a pharmacological approach in parallel. In previous studies, we have confirmed that AR-null mice are ‘functionally similar’ to WT mice treated with an ARI. Nonetheless, we used a whole-body deficient model rather than a cell-specific deficient AR model (e.g., endothelial cell). This, of course, would be a useful tool in this context because we do not know where AR presence contributes most to this phenotype (VSMC vs. endothelial cell, for example). For example, overexpression of human AR (hAR) in the endothelium (and systemically) of an apoE-null mouse accelerates atherosclerosis in a streptozotocin diabetic setting and in an ARI-sensitive manner [36]. Although the STZ model induces a Type I diabetes state of severe hyperglycemia, our HFD model increased blood glucose by approximately 20%, yet this may be sufficient to drive AR-dependent effects in the endothelium. A previous study showed that only 2 weeks of HFD feeding in young male C57BL/6J (WT) mice induced endothelial cell insulin resistance [44], perhaps due to increased circulating fatty acids such as palmitate [45]. How AR contributes to the earliest deleterious changes in endothelial cells that give rise to ED under HFD feeding remains both a mystery and a potential therapeutic target [15].

## 5. Conclusions

AR has long been implicated in the chronic manifestations of hyperglycemia and diabetes, including retinopathy, neuropathy, nephropathy, and cardiovascular diseases [15]. Unfortunately, AR inhibitors are not widespread, yet their potential is great, but it is dependent on further understanding of the role of AR in other conditions such as obesity. These current data indicate that AR mediates ED associated with short-term HFD feeding, but it does not contribute to the observed ‘compensatory function’ of aortic PVAT. These data suggest that the ED of HFD feeding in the short term is AR-dependent. Thus, likely endothelial-localized AR remains a potential target of temporally selective intervention in obesity, diabetes, and perhaps other diseases.

## Figures and Tables

**Table 1:** Physiological parameters of WT and AR-null mice after 4 weeks of HFD feeding, starting either at an age of 8 weeks or 18 weeks old.

Age	12 Weeks Old			
Groups	WT and NC	WT and HFD	AR-Null and NC	AR-Null and HFD
*n*	8	8	5	4
Glucose (mg/dL)	92 ± 11	108 ± 15 *	89 ± 6	97 ± 4 *
Body weight (g)	23.9 ± 0.5	28.8 ± 1.1 *	24.3 ± 0.3	30.0 ± 1.7 *
Gonadal fat pad: BW (%)	1.3 ± 0.1	3.2 ± 0.6 *	2.1 ± 0.1	6.0 ± 1.0 *
Age	22 weeks old			
*n*	6	8	6	8
Glucose (mg/dL)	81 ± 8	102 ± 21 *	81 ± 8	113 ± 16 *
Body weight (g)	24.4 ± 0.5	34.2 ± 2.0 *	27.4 ± 1.0	40.4 ± 1.1 *
Gonadal fat pad: BW (%)	1.3 ± 0.1	6.2 ± 0.7 *	2.5 ± 0.4	7.5 ± 0.3 *

Values = mean ± SE (*n* = 4–8 mice per group); area measurements in mm^2^; Abbr.: WT, wild type; NC, normal chow; HFD, high-fat diet; AR-null, aldose reductase-null; *, *p* < 0.05 vs. genotype-matched NC control.

**Table 2:** Morphometric analyses of murine thoracic aorta and surrounding perivascular adipose tissue (PVAT): effects of 4 weeks of HFD feeding, age, and the absence of aldose reductase (AR-null).

Groups	WT and NC	WT and HFD	AR-Null and NC	AR-Null and HFD
Measurement	12 weeks old			
DAT Area	398,367 ± 76,883	748,735 ± 125,439	481,555 ± 72,726	784,229 ± 161,008
VAT Area	266,800 ± 26,695	386,549 ± 32,881	353,759 ± 45,750	394,109 ± 65,879
PVAT Area	665,168 ± 91,534	1,135,285 ± 143,463	835,314 ± 101,186	1,178,337 ± 222,735
WAT Cluster Area	3459 ± 942	12,163 ± 7042	19,662 ± 7850	21,169 ± 13,925
WAT Cell #	37 ± 3	96 ± 54	167 ± 108	89 ± 58
WAT Cell Area	128 ± 22	198 ± 12 *	206 ± 24	302 ± 60 *
Aorta, X-sec Area	135,781 ± 14,048	156,874 ± 25,033	146,826 ± 18,009	153,411 ± 20,258
	22 weeks old			
DAT Area	461,501 ± 82,989	839,290 ± 191,444	704417 ± 136492	1,084,809 ± 119,937
VAT Area	290,257 ± 41,676	252,562 ± 19,120	254685 ± 115211	500,949 ± 66,649
PVAT Area	751,758 ± 119,178	1,091,852 ± 185,357	959102 ± 21281	1,585,758 ± 149,400
WAT Cluster Area	2983 ± 354	14,909 ± 5043 *	15442 ± 5979	24,003 ± 6708
WAT Cell #	46 ± 5	100 ± 33	158 ± 70	96 ± 1
WAT Cell Area	103 ± 4	402 ± 71 *	158 ± 16	511 ± 93 *
Aorta, X-sec Area	171,875 ± 37,600	200,722 ± 65,029	160,636 ± 1338	152,541 ± 18,914

Values = mean ± SE (*n* = 2–8 mice per group); Area measurements in mm^2^; Abbr.: WT, wild type; NC, normal chow; HFD, high-fat diet; AR-null, aldose reductase-null; WAT, white adipose tissue. *, *p* < 0.05 vs. matched NC control; #, 0.10 > *p* > 0.05 vs. matched NC control.

## Data Availability

Data availability will be provided upon any reasonable request. We prefer to share data upon reasonable request with other investigators in order to foster collaborations.

## Supplementary Materials

The following supporting information can be downloaded at https://www.mdpi.com/article/10.3390/metabo13121172/s1. Figure S1: Effects of short-term high-fat diet (HFD) on thoracic aorta function without and with perivascular adipose tissue (+PVAT).
