# Supplementary material for: Aldose Reductase (AR) Mediates and Perivascular Adipose Tissue (PVAT) Modulates Endothelial Dysfunction of Short-Term High-Fat Diet Feeding in Mice
Source: Metabolites. 2023 Nov 24;13(12):1172. doi: 10.3390/metabo13121172 (PMC10744918; doi:10.3390/metabo13121172)
Supplement: Supplementary file 1 [file metabolites-13-01172-s001.zip › metabolites-2672181-supplementary.pdf]

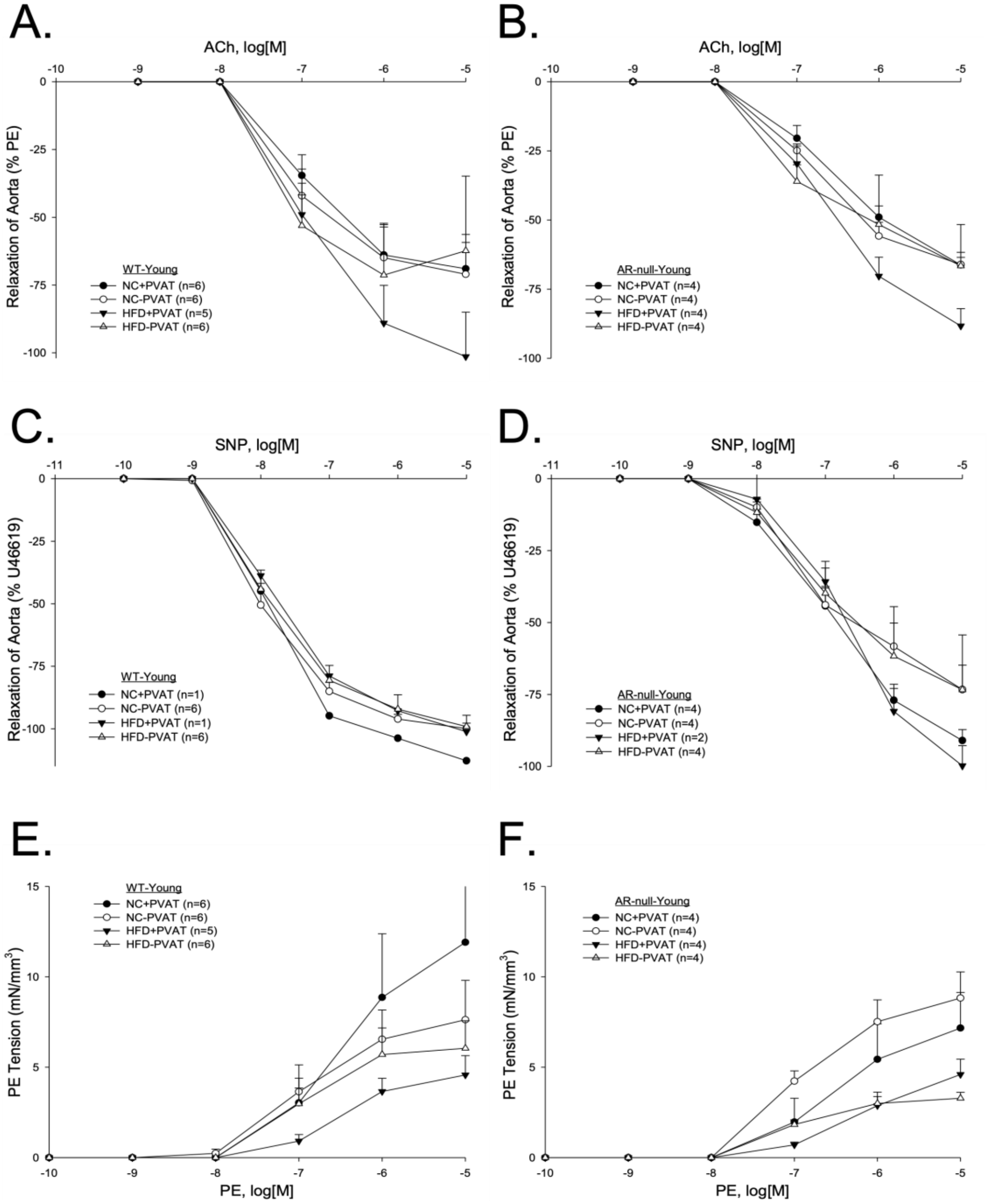

Figure S1. Effects of short-term high-fat diet (HFD) on thoracic aorta function without and with perivascular adipose tissue (+PVAT).
